# The histidine brace: nature's copper alternative to haem?

**DOI:** 10.1002/1873-3468.14579

**Published:** 2023-01-29

**Authors:** Paul H. Walton, Gideon J. Davies, Daniel E. Diaz, João P. Franco‐Cairo

**Affiliations:** ^1^ Department of Chemistry University of York UK

**Keywords:** copper, haem, histidine brace, LPMOs

## Abstract

The copper histidine brace is a structural unit in metalloproteins (*Proc Natl Acad Sci USA* 2011, **108**, 15079). It consists of a copper ion chelated by the NH_2_ and π‐N atom of an N‐terminal histidine, and the τ‐N atom of a further histidine, in an overall T‐shaped coordination geometry (*Nat Catal* 2018, **1**, 571). Like haem‐containing proteins, histidine‐brace‐containing proteins have peroxygenase and/or oxygenase activity, where the substrates are notable for resistance to oxidation, for example, lytic polysaccharide monooxygenases (LPMOs). Moreover, the histidine brace is an invariant unit around which different protein structures exert different activities. Given the similarities in the diversity of function of proteins that contain either the copper histidine brace or haem, *the question arises as to whether the functions of histidine brace‐containing proteins duplicate those containing haem groups.*

## Graphical abstract

The histidine brace and haem groups show similarities between different proteins: structural invariance but both supporting a range of different functions.

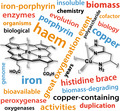

## Abbreviations

**AA**, auxiliary activity

**Bim1**, LPMO‐like protein

**CAZy**, carbohydrate‐active enzyme

**CopC**, copper resistance protein C

**DUF**, domain of unknown function

**GOE**, great oxygenation event

**LPMO**, lytic polysaccharide monooxygenase

**pAMO**, particulate ammonia monooxygenase

**pMMO**, particulate methane monooxygenase

**PmoF1**, periplasmic copper chaperone

**X325**, LPMO‐like protein

**YcnI**, protein from ycn operon in Gram‐positive bacteria

The functional activities of the many known iron‐containing proteins stretch across the full range of biochemical functions, including O_2_ transport, oxygenases, peroxygenases, electron transfer proteins, metal transport proteins and enzymes capable of small molecule activation. These functions are also often found in copper‐containing analogues. Such duplication of activity within biology is antithetical to the usual efficiency of genome evolution, which quickly discards redundant or unnecessary protein synthesis [3].

The commonly accepted wisdom behind the duplication of activities of iron and copper‐containing proteins is that there was a rapid rise in the concentration of O_2_ in Earth's atmosphere between 800 and 500 million years ago. This increase, the so‐called Great Oxygenation Event (GOE), drove two key chemical reactions [4]. The first was the oxidation of soluble Fe(II) in what had been previously largely anaerobic oceans to Fe(III), which – despite the concomitant rises in ${{\mathrm{SO}}_{4}}^{2-}$ concentrations – saw Fe concentrations fall by four orders of magnitude, from ca 10^−5^ to 10^−9^ M. This fall was largely due to the formation of insoluble Fe(OH)_n_(O)_m_(H_2_O)_p_ species, for example, *K*_sp_ of Fe(OH)_3_ ~ 10^−39^ mol^4^ dm^−12^. The same event saw oxidation of Cu(I) to Cu(II) and of sulfide (S^2−^) to sulfates (${{\mathrm{SO}}_{n}}^{2-}$, *n* = 3,4), thus releasing copper from the profoundly insoluble Cu_2_S (K_sp_ ~ 10^−48^ mol^3^ dm^−9^) into dissolved Cu(II). The resulting copper concentrations are estimated to have increased by 12 orders of magnitude over this time, from ca 10^−25^ to 10^−13^ M [4].

Such a shift in the relative concentrations of dissolved Fe and Cu, from a ratio of 10^20^–10^4^ over a period of ~ 100 million years, represented what can only be described as an existential shock to a well‐established biological system, the response of which came in three separate forms. The first was to ‘protect’ the existing biological processes dependent on iron by developing methods through which the reduced levels of bioavailable iron could be sequestered and maintained. Perhaps the most conspicuous of these responses is that of high Fe‐affinity small molecule siderophores secreted by prokaryotes for the acquisition of available Fe, and of the later role of transferrin and ferritin proteins in mammals for the dedicated transport and storage of Fe [3]. The second was to develop means by which the toxicity of higher levels of copper could be managed. Accordingly, there exists an array of Cu import, transport, chaperone and export proteins, especially in the role of Cu in mammalian disease, many of which are active areas of research [5]. The third response was the evolution of what might be called *de novo* Cu proteins that could replace or duplicate the role of iron equivalents. It is this last response that has led to the ostensible duplication of the activities of many Fe and Cu proteins, with some organisms capable of switching between proteins dependent on one metal to the other based on the relative availability of Fe and Cu in the surrounding milieu [6].

## Haem co‐factor

The haem cofactor is employed in many different molecular functions, ranging from essential electron transfer reactions in energy conversion to the oxidative functionalisation of organic molecules [3]. Moreover, in addition to the range of chemistry offered by the haem cofactor, it represents an efficient use of a chemical unit, the essential tetrapyrrole core, which despite being largely invariant can be extensively and readily repurposed by relatively small changes in the periphery of the molecule within a protein matrix. In many ways, the haem group is the epitome of how the chemical potential of a basic molecular unit can be utilised to many different ends – a feature that has been used of late in the repurposing of existing haem‐dependent enzymes towards new activities [7]. While adaptation to oxygenase and peroxygenase activities of any pre‐existing haem‐containing enzymes likely did not emerge until after/during the GOE, it is evident that the decreasing bioavailability of Fe presented an existential threat to any organism that did not have copper equivalents.

The adaptability of the porphyrin unit of haem is also seen in the known metals coordinated by porphyrin and its derivatives in biology, which are Fe (haem), Ni (F430), Co (vitamin B12) and Mg (chlorophyll). Such distribution of these metal‐porphyrin complexes reflects their high concentrations in the primordial oceans (~ 10^−8^ M for Ni and Co) before the GOE. In contrast, there is a conspicuous absence of Zn, Cu and Mn porphyrin groups in biological systems. Indeed, given that Mn concentrations in the oceans broadly matched those of Ni and Co both before and after the GOE, and the good amount of knowledge of the rich chemistry offered by synthetic Mn‐porphyrinoid complexes [8], the reasons why the chemistry of Mn‐porphyrins was not harnessed and the complete lack of any known manganese porphyrin in biology remain open questions. For Cu and Zn, instead, their near absence from biology is best explained not only by the fact that the chemistry of Cu/Zn‐porphyrins is limited, but also by the very low bioavailability of these elements before the OEC.^1^ Moreover, it is evident that biology had developed the porphyrin group and all its manifestations without need to recruit the chemistry of Cu in any of the biological processes it required.

## The copper histidine brace

**Fig. 1:**
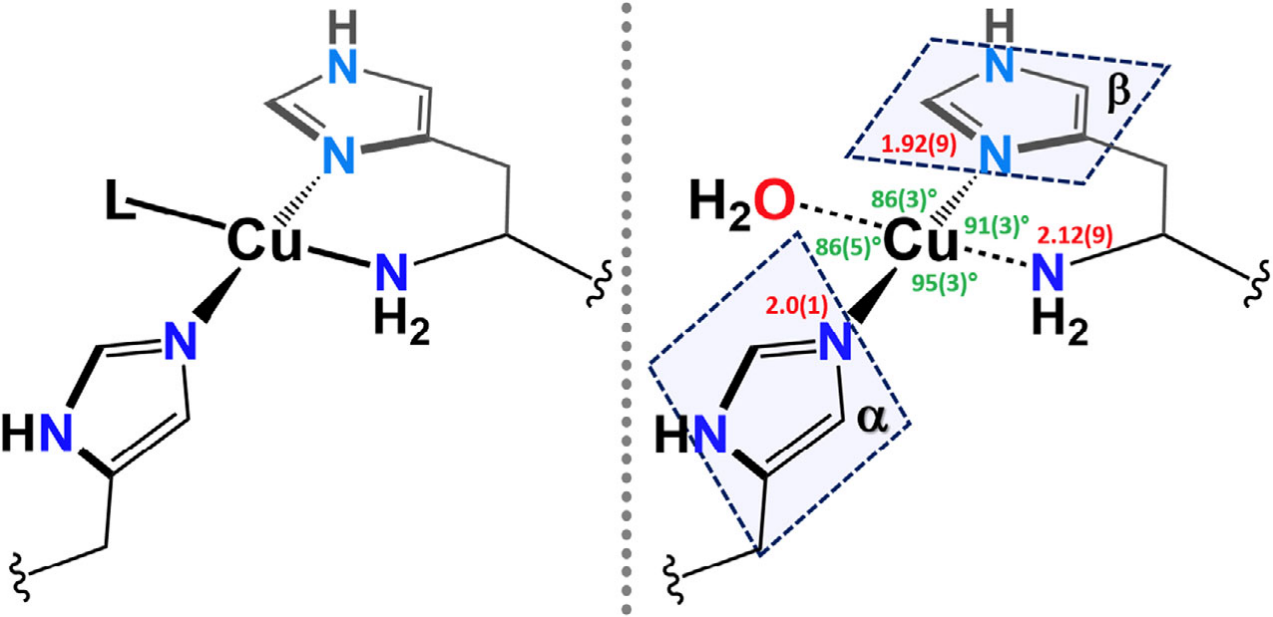
The essential copper histidine brace unit (left), showing average metrical parameters around the Cu and their standard deviations (right). Also depicted are the best‐fit planes (α and β) of the imidazole side chains of the two histidine groups, where the average inter‐planar angle is 60(6)°.

This molecular moiety was discovered in 2011, through spectroscopic and careful calorimetry experiments of the active site of a group of enzymes known as (lytic) polysaccharide monooxygenases [9] ([L]PMOs, Fig. 1) [1, 10, 11, 12, 13, 14, 15, 16, 17, 18]. These enzymes are mostly found in fungal and bacterial organisms that have biomass‐degrading lifestyles. In fact, these very organisms are also the ones that adapted significantly to the rise of plants from ~ 700 million years ago. They responded by equipping themselves with an array of biomass‐degrading (hence ‘lytic’ on polysaccharide chains) enzymes that could utilise the carbon and nutrient sources offered by lignocellulosic biomass. Since the occurrence of plants and increased O_2_ in the atmosphere were contemporaneous, many organisms evolved enzymes that also recruited O_2_ (or H_2_O_2_) to degrade lignin biomass. Notably, in the context of the above‐discussed bioavailability of metals and the iron‐porphyrin cofactor, some of these lignolytic enzymes utilise haem and manganese as cofactors, reflecting the flexibility of the chemistry offered by these groups. In terms of the polysaccharide component of lignocellulosic biomass, however, the often‐high crystallinity of the polysaccharide presented a significant barrier to utilisation of the rich fixed‐carbon source that cellulose offered. This recalcitrance of cellulose and associated polysaccharides, for example, chitin, hemicelluloses, required a new class of enzymes to effect their degradation. It is here that LPMOs and their histidine brace active sites are likely to have entered the genomic record, where organisms evolved a mechanism to overcome the recalcitrance of the crystalline component of different biomasses, and also recruited the now bioavailable Cu as a co‐factor. It is further possible to hypothesise a more precise date of when LPMOs became widespread amongst biomass‐degrading organisms. This date corresponds to the end of the Carboniferous period at ~ 300 million years ago. After this time, most (although not all) biomass was effectively degraded on the surface before it could be lithified, possibly because biomass‐degrading organisms had acquired a new functionality in LPMOs.

The basic unit of the histidine brace (or colloquially ‘His brace’) is simple, akin to the simplicity of the analogous iron‐porphyrin cofactor. The structure is depicted in Fig. 1. It consists of an N‐terminal histidine which chelates a single Cu ion through the nitrogen atoms of the amino terminus NH_2_ and the π‐N atom of the imidazole side chain. This arrangement of atoms gives an overall six‐membered chelate ring, the stability of which can be predicted from the Irving–Williams series to be high in comparison to other M^2+^ transition metal ions. The copper is then further coordinated by the τ‐N atom of the imidazole ring of a further histidine side chain. The overall arrangement of the CuN_3_ unit is T‐shaped at the Cu. In addition to these coordinating groups, in the Cu(II) form of the histidine brace, a further usually exogenous ligand such as water occupies the fourth position *trans* to the NH_2_ coordinating group in an overall equatorial planar coordination. This is true for all LPMOs except for some bacterial ‘AA10’ LPMOs (the CAZy classification [19] of LPMOs is discussed below), where two exogenous ligands occupy positions above and below the equatorial plane. Notwithstanding the variability of the fourth ligand, what is evident, and also part of the wider argument presented in this paper, is that the primary coordination sphere of the histidine brace is consistent across all known LPMO structures. In fact, recent surveys of known structures of LPMOs showed that there was no significant variation in the metrical parameters around the Cu, including a ‘twist’ of ~ 60° between the two best fit planes of the imidazole groups of the brace [2, 20]. In other words, the primary histidine brace structure, especially the N‐terminal coordinating histidine, can be viewed as an essentially fixed unit.

**Fig. 2:**
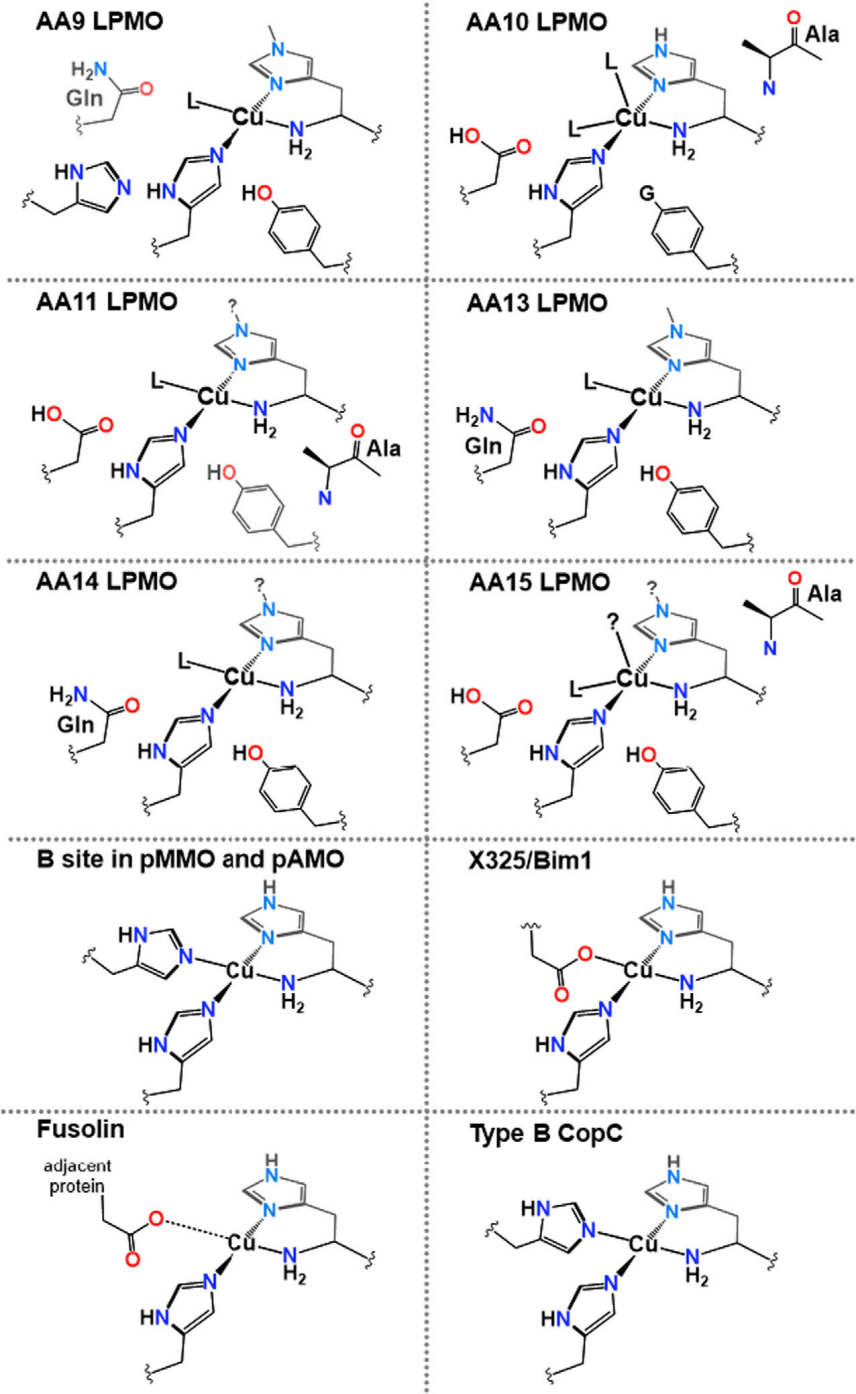
Active site structures of known copper histidine brace active sites, showing protein class name. LPMOs are classified according to the CAZy database (G = H or OH) [19]. Note that some structures exhibit methylation of the τ‐N atom of the histidine brace. The role of this post‐translation modification is unknown, but may be related to the stability of the histidine brace towards deleterious oxidation [24, 25].

The histidine brace occurs not only in LPMOs but also in other proteins. Despite this, the differences in the histidine brace unit in all cases are small. Any existing differences occur between the amino acid side chains that occupy the secondary, and in two cases primary, coordination sphere of the Cu. These variations are depicted in Fig. 2. For instance, the active site of site B in pMMO is shown in Fig. 2, where the nitrogen atom of the side chain of a histidine group takes up the fourth coordination site. Also shown is the active site of a protein with unknown function where the fourth coordination site around the Cu is not occupied with an exogenous ligand, but with the oxygen atom of the carboxylate of a nearby aspartate group (LPMO‐*like* protein X325/DUF6595) [21]. The biochemical roles of both of these sites are currently unknown, although X325 is implicated in Cu transport within fungi, and site B of pMMO was originally believed to be the site where CH_4_ oxidation occurred. The latter of these was recently revised from an original di‐nuclear Cu_2_ structure, partly in light of the fact that mononuclear Cu sites are now known from LPMOs and other studies to be highly active as C‐H oxidation catalysts [2, 22]. Finally, the active site of an LPMO protein, which is found in viral spindles of the fusolin protein present in insect viruses, is also shown in Figure 2 [23]. Here the proteins are packed within a crystal, such that the fourth coordination site of the Cu comes close to an aspartate group of a neighbouring protein. The Cu…O distance of 2.7 Å precludes the formation of a Cu‐O bond through this interaction, but the steric presence of the group certainly hinders access to the copper by any exogenous ligands [13]. Once the protein is dissolved in solution, it appears to become active for the oxidation of chitin.

### The stability of the histidine brace

The low coordination number of the copper in the histidine brace makes the metal kinetically labile such that other ligands can interact with and potentially remove the copper from the protein. Necessarily, therefore, the thermodynamic stability of the copper histidine brace unit is an important factor in its overall function. In this regard, from the Irving Williams series, the relative stability of the copper complex of the histidine brace over other M^2+^ 3d transition metal ions is expected to be higher. This high stability of the Cu(II)‐histidine brace complex appears to be realistic since there are no reports of any other metal binding to the histidine brace in proteins, except from some ostensibly incorrect assignments in early LPMO structures [9], or adventitious complexes with high concentrations of metal ions used in protein purification or present in crystallisation screens [26]. Moreover, the features that would be required for any anti‐Irving Williams binding properties, such as strong ligand donation in the putative axial positions of the copper coordination sphere, are not present in any known structures of the histidine brace in proteins [27]. In other words, the N_3_ coordination geometry of the brace is fixed to coordinating a metal ion within its equatorial coordination positions, leaving the axial positions for only weak interactions with other ligands. It is notable in this regard that several LPMO structures contain what appear to be ‘buttressing’ amino acid side chains (see for example the extra histidine residue in the structure of AA9 LPMO in Fig. 2), which appear to hold the coordinating histidine residues in position, preventing any movement away from coordinating a metal in its equatorial plane. As shown by recent studies of Ni binding to synthetic proteins, this hinders the coordination of metal ions other than Cu [27].

Stability constants of copper–LPMO complexes have been measured in a couple of cases. Following chelation studies with EDTA at pH 5, a Cu(II)‐AA9 LPMO stability constant was originally estimated to be > 10^12^ dm^3^ mol^−1^ [1]. Subsequent isothermal calorimetry studies place the enthalpy of Cu(II) binding at ca 10^11^ dm^3^ mol^−1^ [12]. The Cu(I) stability constant with an AA10 LPMO was also determined to be ~ 10^9^ dm^3^ mol^−1^ [28]. The stability of type B Cu(II)‐CopC is ~ 10^9^ dm^3^ mol^−1^ [29]. Type A Cu(I) ‐CopC and Cu(II)‐CopC were measured to be 10^7^ to 10^13^ dm^3^ mol^−1^ and 10^13(1)^ dm^3^ mol^−1^, respectively (see below) [30]. The overall emerging picture is that the copper histidine brace unit is one of the moderately high thermodynamic stabilities, in both Cu(I) and Cu(II) oxidation states. Indeed, the Cu(I) state seems to be indefinitely stable in the absence of oxidising agents.

**Fig. 3:**
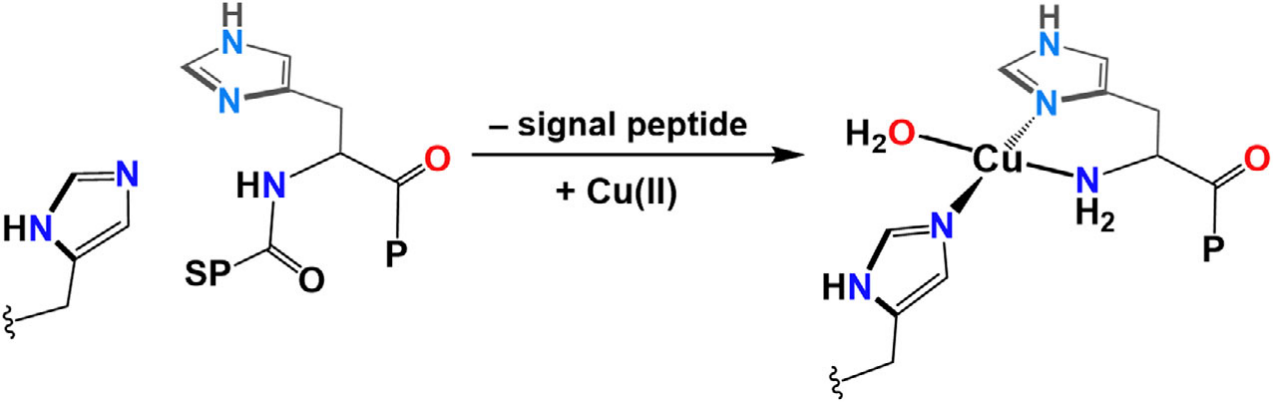
Co‐occurrence of signal peptide removal and coordination of Cu(II). P, protein; SP, signal peptide.

All known LPMO sequences contain N‐terminal signal peptides for secretion into the surrounding milieu or into the periplasm (for Gram negative bacteria), commensurate with a role associated with the degradation of biomass [31], cell wall remodelling, pathogen virulence and organism development [32, 33]. Indeed, beyond LPMOs, all the protein classes shown in Fig. 2 have N‐terminal signal peptides which are cleaved off after secretion. Before cleavage of the signal peptide, the histidine brace unit is unable to bind Cu without deprotonation of the NH amide at the N‐terminus (Fig. 3). This deprotonation will not occur at biological pH values. Therefore, what becomes evident is that cleavage of the signal peptide is commensurate with the ‘demasking’ of the amino terminus NH_2_, such that it can coordinate to a Cu ion. In other words, Cu cannot be coordinated strongly by the protein until the signal peptide has been cleaved. Necessarily, this means that the copper histidine brace is only functional after it has been secreted beyond the membrane, after which it is assumed that there are high enough free Cu concentrations in the surrounding milieu to be coordinated by the histidine brace. Presumably this activation method occurs in some cases to prevent Cu chelation by the protein within the cytoplasm of the cell, where it could potentially react with reducing agents and O_2_ to generate reactive oxygen species.

### Known occurrence of the histidine brace in biology

**Fig. 4:**
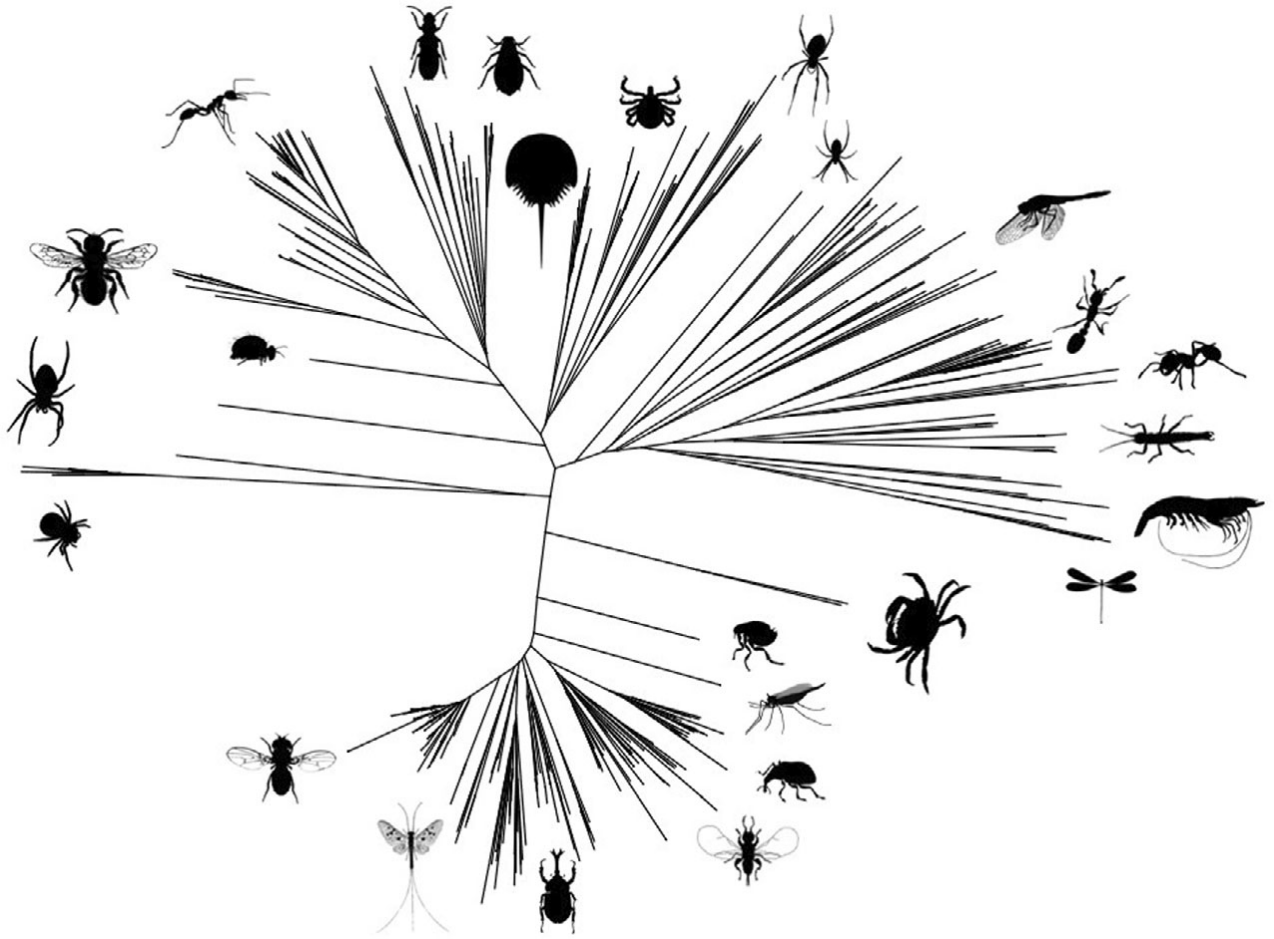
Radial phylogram of AA15 genes across animalia taxa, with example organisms. Phylogram was generated with Dendroscope [38] using sequence alignments based on Uniprot sequence A0A2N8U5I8. Images are public domain (CC0) and were taken from PhyloPic (https://beta.phylopic.org/).

From reported genomic and proteomic sequences, the histidine brace is known to be widespread in biology [34]. For instance, the most authoritative sequence‐based database for LPMOs and other enzymes active on carbohydrate‐containing molecules is the Carbohydrate‐Active EnZYme (CAZy) database [19, 35]. This resource has the advantage over others in that the classifications are manually curated for their known biochemical activity, thus avoiding some of the many errors of sequencing and mis‐annotations that can occur in other databases. Therein, CAZy lists eight different genomic classes of histidine‐brace containing enzymes, all of which have LPMO activities. These different classes are labelled as ‘auxiliary activity’, or ‘AA’ enzymes, where LPMOs fall into classes AA9, AA10, AA11 [11], AA13 [12], AA14 [36], AA15 [15], AA16 [18] and AA17 [34]. These classes also roughly divide into different organismal phylogenies. Fungal organisms dominate the AA9, AA11, AA13 and AA14 classes. Bacterial LPMOs constitute the bulk of the AA10 class. LPMOs derived from the AA15 class are found in viruses, oomycetes, many insects, arthropods, cephalopods and crustacea, amongst others. Indeed, the AA15 class is the most widespread gene of LPMOs, extending deep into the Animalia kingdom, including *Drosophila melanogaster* (fruit fly), *Limulus polyphemus* (horseshoe crab), various crustaceans, octopodes and many spiders (Fig. 4; Table 1) [15]. The histidine brace also appears in Bim1, a known Cu binding protein that occurs associated with other copper transport proteins in fungi [37]. The protein is membrane‐bound via a GPI anchor on fungal cell walls.

### Modifications to the primary coordination sphere of the histidine brace

**Fig. 5:**
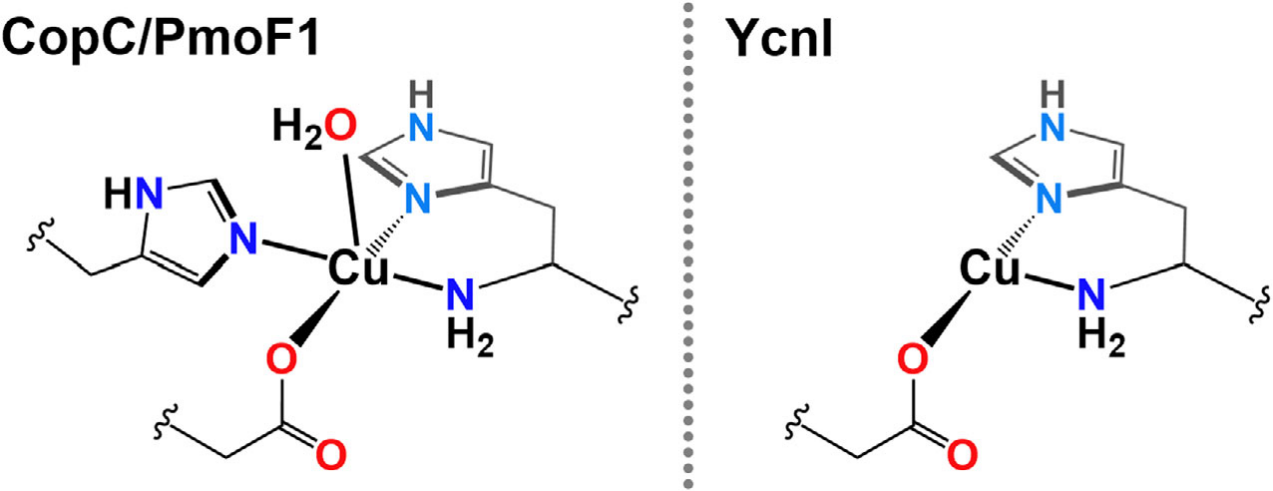
Histidine brace‐like active sites in CopC and PmoF1 (PDB codes: 5ICU and 6P16) and YcnI (PDB code 1MEK). It is presumed that the YcnI site has copper in the Cu(I) oxidation state, possibly due to photoreduction during data collection.

As first described in 2011 [1], the ‘brace’ moniker represents both the fact that the two histidine groups figuratively *embrace* the copper ion and that there are two (or a *brace* in old English) histidine groups involved in the coordination. Since its original naming, it is becoming evident that the brace can be modified in one of two separate ways. The first is that the position of the NH_2_ group relative to the coordinating N atoms of the histidine groups can vary to be *cis* to one and *trans* to the other. These inorganic forms of position isomers are found in what are thought to be Cu transport proteins in methanotrophs (Fig. 5), such as the Cu(II) binding site in PmoF1 [39]. The second variation is the replacement of the second histidine with another coordinating amino acid side chain. This has recently been shown for the YcnI proteins (DUF1775), thought to be involved in copper transport in bacteria [40]. A single structure of an example of these proteins is available, in which the authors termed the coordination geometry at the copper as a ‘mono‐histidine brace’ (Fig. 5).

**Table 1:** Classes of organisms with genomic copies of histidine‐brace‐containing proteins, along with example organisms.

Protein (CAZy entries)	Highest classification	Next highest (most populated)	Example organisms (common names)
AA9 (1017)	Dikarya	Pezizomycotina Basidiomycota	*Aspergillus niger* (black mould) *Coprinopsis atramentaria* (common inkcap)
AA10 (8753)	Bacteria (and some baculoviridae)	Proteobacteria Terrabacteria	*Listeria monocytogenes* (Listeria)
AA11 (274)	Opisthokonta	Dikarya Pezizomycotina Basidiomycota	*Aspergillus oryzae* (kōji fungus)
AA13 (38)	Pezizomycotina	Leotiomyceta	*Fusarium graminearum* (fusarium ear blight fungus)
AA14 (53)	Opisthokonta	Dikarya	*Trametes coccinea* (southern cinnabar polypore)
AA15 (363)	Eukaryota Phycodnaviridae	Bilateria Chlorovirus	*Limulus polyphemus* (horseshoe crabs) *Galendromus occidentalis* (western predatory mite)
AA16 (82)	Dikarya	Leotiomyceta	*Botrytis cinerea* (botrytis bunch rot)
AA17 (421)	Oomycota	Saprolegniaceae	*Phytophora infestans* (potato late blight fungus)
pMMO pAMO	Bacteria	Proteobacteria	*Methylocystis* (methanotrophs)
X325/Bim1	Agaricomycetes	Multiple fungal species	*Trametes versicolor* (Turkey tail fungus)

These structures of histidine brace derivatives are intriguing from a functional point of view. The majority seem to be associated with copper transport in bacteria, often appearing on the same gene cluster of other copper transport proteins. There have been proposals that these proteins, along with X325, could demonstrate redox activity similar to LPMOs, but none has convincingly been shown, except for some weak ascorbate oxidation activity on mutants of CopC. Accordingly, it appears as if this group of proteins is associated with Cu transport functions, although some recently reported redox activity of CopC may indicate a more functional protein [30, 41].

## Parallels between known activities of haem‐containing proteins and those containing the histidine brace

In light of the discussion above, it becomes an interesting question as to what known functions of haem‐containing proteins also occur in proteins with the copper histidine brace. Table 2 lists the major known biochemical functions of haem proteins and a comparison with known functions of proteins containing the histidine brace. It can be seen that the oxygenase, peroxygenase and catalase activities are shared between the two classes. Such powerful oxygenases constructed from such simple units as the histidine brace and the haem group is a measure of the efficiency of biological systems to harness basic chemistry offered by a molecular unit to perform a variety of different tasks. In fact, the ability of both classes to bind and activate O_2_ or H_2_O_2_ is a defining feature common to both. It is here where the biology seems to have recruited the histidine brace most effectively, in extracellular oxidation, making use of three factors: the availability of O_2_, the higher concentrations of soluble copper and Irving‐Williams series that dictate that copper is selectively chelated by histidine brace proteins.

**Table 2:** Comparison of the broad functions of known haem‐containing and histidine‐brace‐containing proteins.

Known activity of haem‐containing proteins	Histidine brace equivalent
Globins: O_2_ transport/storage (e.g. haemoglobin, myoglobin, neuroglobin)	Unknown
Oxygenase (e.g. P450)	All known LPMOs. Some reports of activity in CoC‐like proteins
Peroxygenase (e.g. lignin peroxidases)	Some LPMOs
Oxidase and peroxidase	Known for LPMOs interacting with electron‐donor proteins, but in the absence of polysaccharide substrate
Electron transfer (cytochrome *c*, cyt *c* and cytochrome *b*_5_, cyt *b*_5_)	Unknown, but potential role for X325 or YcnI?
Catalase	Known for some LPMOs
O_2_ reduction (e.g. cytochrome *c* oxidase)	A characteristic reaction of LPMOs in the absence of substrate
Fe transport?	Cu transport
NO transport	Unknown
Reductase (e.g. cytochrome *cd*_*1*_ nitrite reductase)	Unknown

It is also evident that the known activities of copper histidine brace proteins do not, as yet, cover the full range of haem‐protein functions. Necessarily the reasons for this are that these functions may not have been discovered or that the role of histidine brace‐containing proteins is restricted to activities outside the cytoplasm of the organism's cells. This latter aspect is in accordance with the fact that all known histidine brace proteins carry a signal peptide for their secretion, which would argue that all histidine brace proteins are extracellular [19]. However, it is also evident from studies of the position‐specific propensities of amino acids in protein sequences, that an N‐terminal histidine is a common feature of many proteins, secreted or not. And while the current CAZy database [35] does not show any known LPMOs in the human genome (see http://www.cazy.org/e355.html), such a group will always be capable of chelating a transition metal ion, and – according to the Irving–Williams series – in most cases a Cu(II) ion will be chelated in preference to all others. It is to be expected, therefore, that more Cu(II)‐containing cytoplasmic proteins where the copper is bound to an N‐terminal histidine will be discovered, beyond those shown in Table 2. Whether these have functions beyond copper transport remains to be determined. As for the other functions, however, the latent redox capacity of the histidine brace would suggest that these functions are at least plausible if not likely and may well yet emerge as more N‐terminal histidine proteins are characterised.

### The nature of the oxidising intermediate

Given the parallels in the abilities of haem‐containing and histidine‐brace containing oxygenases to catalyse the oxidation of strong C‐H bonds by O_2_, it is instructive to compare the reactive species in the catalytic cycles of both. In this regard, much is known about Compound I in P450 monoxygenase, which is a Fe(IV) = O‐radical cation porphyrin complex that can effect the transfer of a hydrogen atom from the C‐H bond in the substrate [42]. This species principally derives its catalytic power from the basicity of the Fe = O group, which is necessarily a function of the bonding between the oxygen atom and the Fe. Herein, a multiple‐bond can form between Fe and the oxygen atom reflecting the low d‐electron count of the Fe and its ability to accept donation of π‐electron density from the oxygen atom. In contrast, the analogous species that can form at the histidine brace, a copper‐oxyl [Cu‐O]^+^, is unable to form such π‐bonds due to the high d‐electron count of the Cu and the ‘oxo‐wall’: This is a fundamental difference between the haem group and the histidine brace [43]. Understanding how this difference still affords similar reactivity between the two groups will depend on a clearer insight into the electronic structure of any [Cu‐O]^+^ unit that might form within the histidine brace, although this species is yet to be observed in the condensed phase. Accordingly, its trapping and subsequent spectroscopic study is a key objective in understanding how the copper histidine brace is capable of catalysing the oxidation by O_2_ of strong C‐H bonds (~ 100 kcal·mol).

## Conclusions

The Great Oxygenation Event, 1.5 billion years ago, forced Nature to adapt. Amongst the many changes that ensued, one was the greatly increased use of Cu in proteins, to duplicate and replace the well‐established Fe‐dependent chemistry. The result was the emergence of new copper‐dependent proteins and a duplication of the activity of certain iron‐containing proteins. Of the iron‐containing molecular units which have emerged from evolution, the haem group perhaps offered the greatest challenge in terms of finding a copper alternative. This challenge stemmed from the adaptability that the haem group and its chemistry offered to biology – a wide range of biochemical functions from a single unit. Could this adaptability of a single unit be replicated with a copper‐containing group? The answer appears to be yes in the form of the copper histidine brace, which – like haem – is an essentially fixed structural unit that adapted to a variety of roles. The basic function of the unit is modified by the surrounding amino acids of the protein structure. Moreover, as porphyrin can be adapted to various porphyrinoids, some modifications to the histidine groups of the histidine brace also appear to be possible, for example, methylation of the N‐atom of the N‐terminal histidine. This tactic is undoubtedly one that is resource efficient from both chemical and energy perspectives. The open question is what other chemistry and biochemical function will emerge for proteins containing the copper histidine brace.

## Author contributions

PHW and GJD conceived of the concept. All authors contributed to the writing of the manuscript.

## Endnote

^1^
Copper‐uroporphyrin III is a naturally occurring pigment found in feathers. Copper‐porphyrins have also been found in deep‐sea sediments. Whether these trace compounds have a biochemical function or not is far from certain.
